# *Lactococcus garvieae* endocarditis presenting with subdural haematoma

**DOI:** 10.1186/1471-2261-14-13

**Published:** 2014-02-01

**Authors:** Magnus Rasmussen, Josefin Björk Werner, Mikaela Dolk, Bertil Christensson

**Affiliations:** 1 Division of Infection Medicine, Lund University, From the Department of Clinical Sciences, BMC, B14, Tornavägen 10, Lund 22184, Sweden

**Keywords:** Infective endocarditis, Lactococcus garvieae, Subdural haematoma

## Abstract

**Background:**

*Lactococcus garvieae* is a rare cause of infective endocarditis (IE) in humans and the bacterium can easily be misidentified. Intracranial haemorrhage often occurs in conjunction with IE, but subdural haemorrhage (SDH) is very rarely encountered.

**Case presentation:**

The patient was an 81-year-old male with a history of cardiovascular disease and a prosthetic biologic aortic valve. He presented with fatigue and an acute onset of headache. Computed tomography (CT) revealed a left-sided fronto-temporal subdural haematoma. Low-grade fever was noted and blood cultures yielded growth of *L. garvieae.* Transesophageal echocardiography (TEE) revealed small vegetations on the native mitral valve and on the prosthetic aortic valve. Treatment with penicillin and tobramycin was initiated and the recovery was slow but uneventful.

**Conclusion:**

This is the first report of a case where SDH was the sole presenting neurological sign of IE. The case demonstrates that IE should be considered in patients with SDH where a history of trauma is absent, especially if the patient has fever or predisposing conditions such as a prosthetic heart valve.

## Background

*Lactococcus* is a Gram-positive, catalase-negative bacterium, growing in pairs or in short chains. Lactococci can easily be mistaken for enterococci based on similarities in biochemical reactions [1]. The first report of infective endocarditis (IE) caused by *Lactococcus sp.* was published in 1955 [2], and since then a small number of IE cases have been reported with *Lactococcus lactis*[3] and *Lactococcus garvieae*[4]. Infections caused by *L. garvieae* have been proposed to be mainly transmitted to humans from contaminated fish [5], and IE seems to be a common presentation of infections with *L. garvieae*[6]*.* The infections most often affect prosthetic valves but also native valve IE has been reported [4]. Neurologic complications occur in a subset of patients with IE and embolization leading to ischemia is the most common manifestation [7,8]. Using magnetic resonance imaging (MRI) it has become evident that also haemorrhages occur in at least 10% of patients with IE [9,10]. Haemorrhages are most often intraparenchymal or subarachnoidal [7-10]. Here we report a case of IE where the presenting neurological signs were due to a subdural haemorrhage (SDH).

## Case presentation

**Figure 1:**
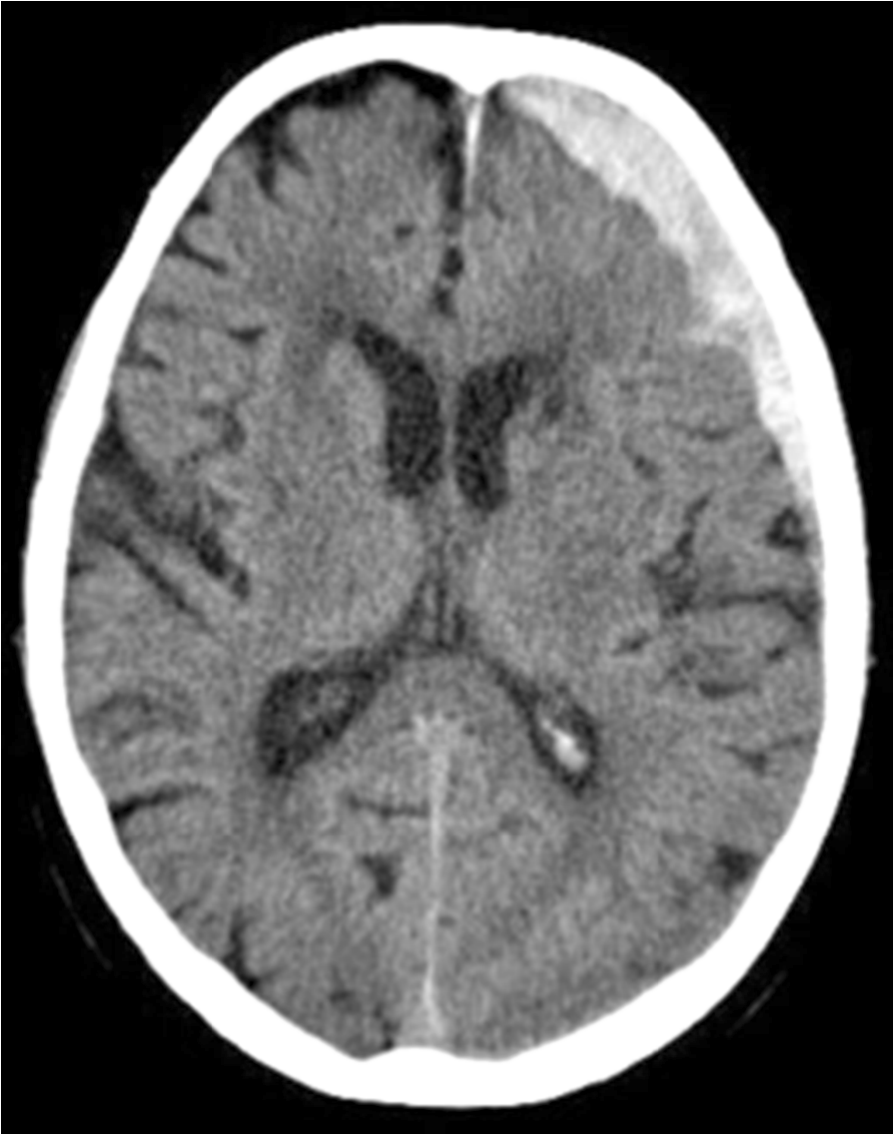
CT scan demonstrating the fronto-temporal subdural haematoma over the left hemisphere.

An 81-year-old male was admitted to a regional hospital with malaise for several months. He had a long history of cardiovascular disease, with multiple myocardial infarctions, coronary arterial bypass grafting, a stent in the left carotid artery, and a prosthetic biologic aortic valve for two years (a bovine, Sorin Soprano valve). The patient was on warfarin treatment due to an atrial fibrillation. Ten months prior to the admittance, the patient experienced an episode of bleeding from a rectal diverticulum. On the day prior to admittance the patient suffered an acute headache and expressive dysphasia. Brain computed tomography (CT) revealed a left-sided 13 mm acute fronto-temporal subdural haematoma (SDH) (Figure 1). The patient denied any form of trauma to the head. Neurosurgeons recommended conservative treatment. PK-INR was 2.1, warfarin was discontinued and vitamin K was administered. Since C-reactive protein was around 50 mg/L and there was a low grade fever (maximum 38.2°C), blood cultures were collected. All four blood culture bottles drawn yielded growth of a Gram-positive coccus identified as *L. lactis* (91% with Vitek II GP). Sequencing of 500 base pairs of the gene encoding 16S rRNA as described in [11], however, revealed 99–100% identity to several sequences from *L. garvieae* and only 85–89% identity to *L. lactis*. A transthoracic echocardiography could not visualize vegetations or other abnormalities.

**Figure 2:**
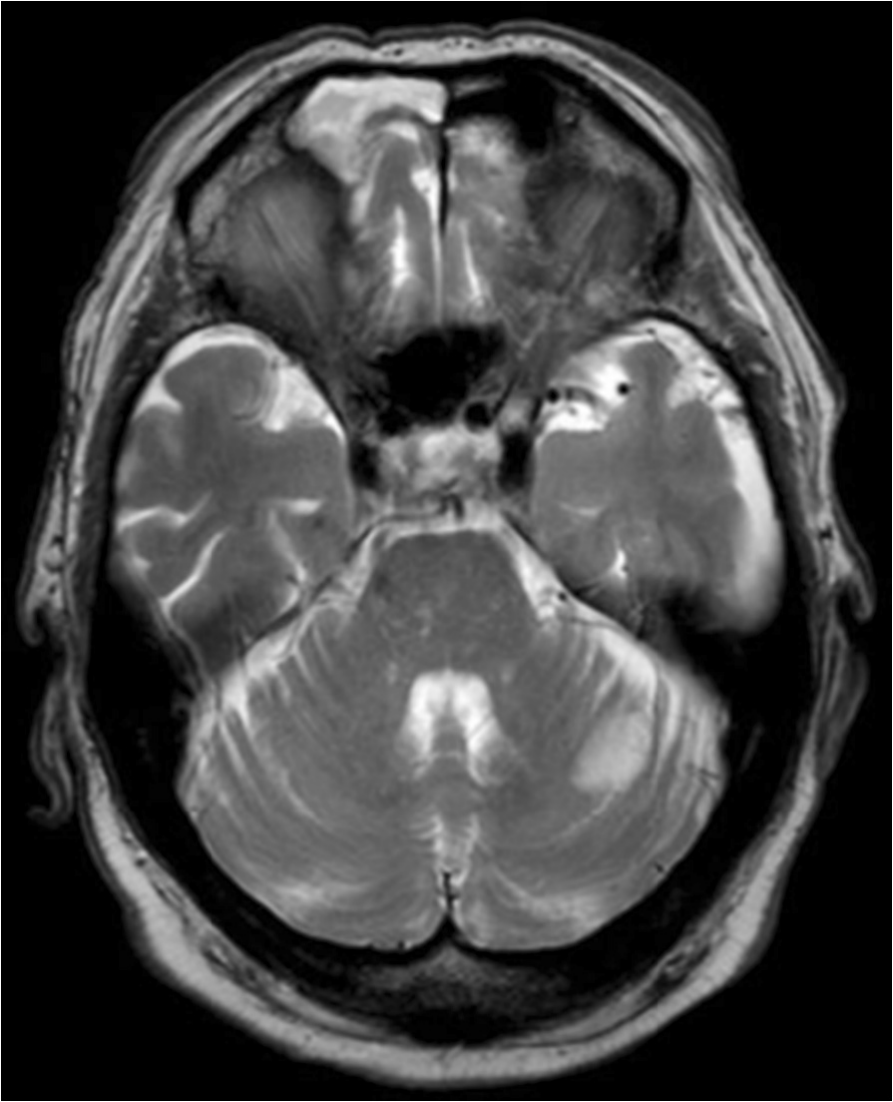
T2-weighted MRI showing the infarction in the left cerebellar hemisphere.

The patient was transferred to the clinic for infectious diseases at Skåne University hospital in Lund after six days and on physical examination he was not dysphatic and had a body temperature of 36.3°C. A systolic murmur with punctum maximum at the left fourth intercostal space was heard. No splinter haemorrhages, Osler nodes, or Janeway lesions were observed. CRP was 118 mg/L and the white blood cell count was 9.1×10^9^/L. A transesophageal echocardiography (TEE) revealed small vegetations on the native mitral valve and on the prosthetic aortic valve. MRI revealed a 1.5 cm large ischemic area in the cerebellum (Figure 2), which could not be seen in the CT performed nine days earlier.

Treatment with penicillin (3 grams, *fid*) and tobramycin (60 mg, *tid*) was initiated. MIC was 0.5 mg/L for penicillin and 2 mg/L for tobramycin. After three weeks of treatment, the patient developed vertigo and hearing impairment, probably resulting from the tobramycin treatment, which was discontinued. The patient slowly became afebrile and CRP gradually decreased. A follow up TEE after three weeks of treatment, showed regression of the assumed vegetation on the aortic valve whereas the mitral vegetation was unchanged. At the request of the patient, no further TEE was performed. After four weeks treatment, a CT-scan showed no signs of additional embolizations and regression of the acute lesion, thus antibiotic treatment was terminated.

The recovery was slow but uneventful. There has been no relapse during 36 months of follow-up.

## Conclusions

IE caused by *L. garvieae* is a rare event and has never been reported from the Scandinavian countries. Our case is the only one reported to the Swedish registry for IE, which comprises 5000 cases of IE. The patient reported that he rarely consumed fish and the mode of bacterial entry into the bloodstream is thus obscure. The case fulfils the modified Dukes criteria for IE [12] since five minor criteria (predisposing heart condition, vascular event, microbiological finding, echocardiographic finding, and fever) were present. The bacteremia in this case does not formally fulfill demands for a major criterion, as lactococci are not listed as typical pathogens [12]. However, from the available cases of lactococcal bacteremia described, it seems that IE is a common presentation of *L. garvieae* infection. In our case, the Vitek II erroneously identified the bacterium as *L. lactis* and correct identification was obtained through sequencing of the 16S rRNA gene. Lactococci can be misidentified as enterococci due to problems with phenotypic methods [1,6,13], therefore the incidence of lactococcal IE may be higher than reported in the literature. Useful biochemical tests for lactococcal identification are given in a comprehensive review [1].

The patient denied trauma to the head and CT did not demonstrate any indirect signs of trauma, making a traumatic cause of the SDH observed in our patient unlikely. The unspecific symptoms of IE preceded the symptoms induced by the SDH, which indicates that the SDH was caused by the IE. Neurological complications are common in IE and have also been described in several cases of lactococcal IE [3,14-17]. Subarachnoidal haemorrhages (SAH) are seen in around 1% of IE-cases and the pathogenesis is believed to involve rupture of a mycotic aneurysm, although such an aneurysm is not always identified [18]. Rupture of mycotic aneurysms can also lead to SDH on rare occasions [19,20]. SDH as a feature of IE is extremely rare, and we have only found three reports of patients with IE complicated by SDH [19,21,22]. We do not believe that the SDH is specifically linked to *L. garvieae* but is rather an uncommon manifestation of IE with any bacterium*.* In the cases of IE induced SDH other types of intracranial haemorrhage also occur. In our case, the SDH was the only intracranial bleeding but the patient also suffered a thromboembolic stroke of the cerebellum later in the course of disease. We did not perform an angiography of the brain so we can neither exclude nor confirm that the SDH was due to a mycotic aneurysm, although this seems to be the most likely pathogenic mechanism. Our case demonstrates that IE can present as SDH and that IE should always be suspected in patients with SDH and concomitant heart valve disorders and signs of infection.

## Consent

Written informed consent was obtained from the patient for publication of this case report and any accompanying images. A copy of the written consent is available for review by the Editor of this journal.

## Competing interests

The authors declare that they have no competing interests.

## Authors’ contributions

MR, JBW, and MD gathered patient data, BC treated the patient and contributed to the conception of the report, MR wrote the report and JBW, MD, and BC assisted with critical revision. All authors gave their final acceptance to the submission of this report. All authors read and approved the final manuscript.

## Pre-publication history

The pre-publication history for this paper can be accessed here:

http://www.biomedcentral.com/1471-2261/14/13/prepub
